# Deep Vein Thrombosis after Lithium Toxicity: A Report of Two Cases and Literature Review

**DOI:** 10.1155/2021/9934037

**Published:** 2021-06-22

**Authors:** Rintaro Sogawa, Shuko Tobita, Akira Monji, Toru Murakawa-Hirachi, Yoshito Mizoguchi, Yuichiro Sakamoto, Hiroyuki Irie, Chisato Shimanoe

**Affiliations:** ^1^Department of Pharmacy, Saga University Hospital, Saga, Japan; ^2^Department of Psychiatry, Faculty of Medicine, Saga University, Saga, Japan; ^3^Department of Emergency Medicine, Saga University Hospital, Saga, Japan; ^4^Department of Radiology, Faculty of Medicine, Saga University, Saga, Japan

## Abstract

Lithium administration can reportedly cause toxicity, including lithium-associated thrombosis; however, not all reported cases of this adverse effect have been attributable to lithium overdoses. We report here two cases of deep vein thrombosis that occurred in association with lithium toxicity. Lithium overdose was deemed to be the cause in only one of these cases; a patient in whom deep vein thrombosis occurred 11 days after identification of lithium toxicity. In the other patient, the deep vein thrombosis occurred 15 days after diagnosis of lithium toxicity; this patient was not considered to have been overdosed. Both patients had other risk factors in addition to receiving lithium. We recommend monitoring D-dimer concentrations to facilitate early detection of deep vein thrombosis in patients with lithium toxicity.

## 1. Introduction

Despite sometimes causing toxicity, lithium is a widely used first-line maintenance treatment for bipolar disorder [1–3]. Symptoms associated with mild toxicity include lethargy, drowsiness, hand tremor, muscle weakness, nausea, vomiting, and diarrhea [4]. Severe toxicity, which can be life-threatening, is associated with grossly impaired consciousness, increased deep tendon reflexes, seizures, syncope, renal insufficiency, coma, and death [4].

Deep vein thrombosis (DVT) is defined as the formation or presence of a thrombus in the deep veins. The causes of venous thromboembolism, including DVTs, can be divided into two groups: inherited and acquired. Many patients have multiple causes [5]. Hereditary risk factors for thrombosis include deficiencies of protein S, protein C, and antithrombin [5]. Acquired risk factors for thrombosis include a prior thrombotic event, recent major surgery, presence of a central venous catheter, trauma, immobilization, malignancy, pregnancy, use of oral contraceptives or heparin, myeloproliferative disorders, antiphospholipid syndrome, and a number of other major medical illnesses [5]. Many patients with an episode of venous thromboembolism have more than one acquired risk factor for thrombosis [6].

Some cases of thrombosis associated with lithium use have been reported. Wasay et al. [7] reported superior sagittal sinus thrombosis associated with lithium-induced nephrogenic diabetes insipidus. Kamijo et al. [8] reported a patient with dural sinus thrombosis and severe hypernatremia that had developed after long-term lithium therapy. DVTs associated with lithium toxicity have also been reported [9]. These reports, however, do not include patients who have taken an overdose (OD) of lithium.

Recently, we investigated lithium toxicity by comparing patients being treated with lithium who did and did not take lithium ODs [10]. We here present two patients who had lithium toxicity and developed DVTs.

## 2. Case Presentations

### 2.1. Case 1

A 31-year-old woman who had been treated at another institution for bipolar disorder was rushed to our hospital because of a drug OD. Her level of consciousness was E3V3M5 on the Glasgow Coma Scale. She had been treated with a combination of lithium (600 mg/d), risperidone (1 mg/d), quetiapine (50 mg/d), valproic acid (600 mg/d), lorazepam (1.5 mg/d), brotizolam (0.25 mg/d), zopiclone (7.5 mg/d), and biperiden (2 mg/d) and had overdosed on all of these drugs (lithium 7200 mg, risperidone 12 mg, quetiapine 600 mg, valproic acid 7200 mg, lorazepam 18 mg, brotizolam 3 mg, and zopiclone 90 mg). On admission, her lithium concentration was 3.95 mEq/L. She had been maintained on lithium for 9 years, her usual lithium concentration being approximately 1 mEq/L. She had no history of thrombosis.

Hemodialysis was performed on the day of hospitalization, followed by continuous hemodiafiltration until Day 4. She was only exposed to a high lithium concentration (≥1.5 mEq/L) for 1 day, after which her lithium concentration rapidly decreased to within the recommended range (Table 1). Intermittent pneumatic compression to prevent DVT was started on the day of hospitalization. She was intubated on admission and extubated on Day 4. She had no nausea or vomiting. A central intravenous catheter inserted on admission was removed on Day 6. On Day 8, she was diagnosed with malignant syndrome based on development of fever, ankylosis, and increased creatinine kinase. Hence, she was reintubated and another central intravenous catheter was inserted. Aspiration pneumonia was also suspected, prompting commencement of antibiotics. Her D-dimer concentrations gradually increased. On Day 11, despite the absence of swelling and color changes, computed tomography of the chest and abdomen revealed numerous thrombi around the catheter in the inferior vena cava, in response to which rivaroxaban (15 mg/d) was added to her treatment regimen. After admission, she was mainly confined to bed. Rehabilitation began on Day 25, after which she gradually increased ambulation. As to psychiatric symptoms, at the beginning of her hospitalization, she was incoherent and acting irrationally; this was resolved over time. An inferior vena cava filter was inserted on Day 19 and removed on Day 36 after confirmation of resolution of her thrombi. She was transferred to another hospital on Day 42 while continuing rivaroxaban.

### 2.2. Case 2

A 66-year-old woman had undergone total knee arthroplasty 1 month previously at our hospital. She had no history of thrombosis. She had previously been treated with a combination of lithium (1000 mg/d), quetiapine (50 mg/d), and flurazepam (15 mg/d) for bipolar disorder and simvastatin (5 mg/d) for hyperlipidemia. She has been maintained on lithium for at least 2 years. At the time of the knee surgery, her lithium concentration was unknown; however, her creatinine clearance (Cockcroft & Gault equation) was 63 mL/min. Postoperatively, graduated compression stockings were used, edoxaban (15 mg/d) was prescribed for 2 weeks to prevent venous thromboembolism, and she was transferred to another hospital for rehabilitation. She was subsequently readmitted to our hospital because of disordered consciousness and lithium toxicity (lithium concentrations 8 days and 1 day before readmission were 1.92 and 1.5 mEq/L, respectively) (Table 1). Her level of consciousness was E4V5M6 on the Glasgow Coma Scale. During rehabilitation, she had been taking nonsteroidal anti-inflammatory drugs. She had no nausea or vomiting while her lithium concentrations were high.

Following readmission, her lithium concentration decreased on fluid replacement therapy alone, without hemodialysis or continuous hemodiafiltration. She was exposed to high lithium concentrations (≥1.5 mEq/L) for 8 days before readmission but was able to eat normal meals. Although her D-dimer concentration was high on Day 2, no thrombi were detected by lower extremity venous ultrasonography. Rehabilitation was resumed on the same day, despite the fact that she was dehydrated and had hypernatremia. Because the delirium that had been apparent at the time of readmission had worsened, asenapine was started on Day 3. On Day 7 (15th day after identification of lithium toxicity), her D-dimer concentration remained high, and lower extremity venous ultrasonography revealed DVTs at the level of both knees. There was no swelling or color changes. The placement of an inferior vena cava filter was considered unnecessary, and apixaban (10 mg/d) was started. Resolution of the thrombi was identified on Day 13. She was discharged on ongoing apixaban on Day 17 for outpatient follow-up.

## 3. Discussion

Lithium toxicity contributed to the development of DVTs in these two patients (11 and 15 days after identification of lithium toxicity). Only the former patient had taken a lithium OD. No hereditary risk factors for DVT were identified in either patient; however, both had some acquired risk factors. Case 1 had undergone insertion of a central intravenous catheter, engaged in prolonged bed rest while being sedated, and been administered antipsychotic agents. She had also developed an infection. Case 2 had undergone total knee arthroplasty and was 66 years old, dehydrated, and taking antipsychotic agents. The presence of multiple acquired risk factors is consistent with previous reports [6]. Because total knee arthroplasty is associated with a high risk of DVT [11], Case 2 was prescribed edoxaban, fitted with compression stockings, and started rehabilitation after readmission to minimize the risk of DVT. In Case 1, intermittent pneumatic compression was started from the day of hospitalization. Nevertheless, both patients developed DVTs after being diagnosed with lithium toxicity, suggesting that this drug may have contributed to the development of that complication. Sedation and the inactivity associated with lithium intoxication can result in impairment of venous return, increasing the risk of developing a DVT. Additional studies are needed to determine whether lithium is an equivalent or greater risk factor than other established risk factors for the development of DVT.

Although we identified DVTs after lithium toxicity, thrombosis has also been reported in patients with normal lithium concentrations. Kamijo et al. [8] reported dural sinus thrombosis despite a normal lithium concentration. An Italian clinical trial of lithium for amyotrophic lateral sclerosis was discontinued because of its lack of efficacy and development of serious adverse events (4.7% of the patients had DVTs) [12]. In the present trial, the lithium concentration was controlled to 0.2–0.8 mEq/L, indicating that, even at nontoxic concentrations, lithium may be associated with the development of thrombosis. Previous case reports have proposed that dehydration and hypernatremia caused by nephrogenic diabetes insipidus can cause lithium-induced thrombosis [7, 8]; however, the mechanism has not been clarified. Renal dysfunction caused by concomitant medications or dehydration is also reportedly a risk factor for lithium toxicity without an OD [13–15]. Dehydration is a well-recognized risk factor for both DVTs and lithium toxicity. However, whether lithium toxicity or normal lithium concentrations are more likely to be associated with DVTs has not yet been determined. As mentioned in the introduction, we have compared the rates of lithium toxicity in patients receiving lithium who did and did not take ODs of this drug [10]. In an additional investigation for the present study, we found that two of 16 patients with lithium toxicity (12.5%) developed thromboses. This is a higher percentage than was reported for the Italian clinical trial. Although thrombosis can occur with normal lithium concentrations, it may be more prevalent in patients with lithium toxicity. This possibility needs to be investigated in larger studies.

After recognizing lithium toxicity, we measured D-dimer concentrations frequently in both of our cases, possibly resulting in early diagnosis of their DVTs. However, D-dimer is a nonspecific marker, high concentrations being found in many conditions (e.g., malignancy, sepsis, recent surgery or trauma, pregnancy, and renal failure). Conversely, high D-dimer concentrations are found in nearly all patients with acute DVTs [16]. Thus, regular screening for, and thorough assessment of, DVTs is recommended for psychiatric patients, especially those who are being restrained [17]. Patients taking lithium who also have other risk factors for DVTs require regular D-dimer monitoring.

Although lithium has been used as a maintenance treatment for bipolar disorder for many years, recent data [18, 19] have shown that it is less effective for this purpose than quetiapine and other atypical drugs. This finding, along with lithium's well-known toxicity, has led to increased criticism of its use, which has resulted in a decline in lithium usage in the USA [20]. Reduction of lithium use will likely result in lower rates of lithium toxicity and associated thrombosis.

## 4. Conclusions

We here report two cases of DVT associated with lithium toxicity. DVTs may develop even in patients taking lithium as prescribed. Regular monitoring of lithium concentrations and renal function is essential during lithium use. We also recommend taking care to maintain adequate hydration and monitoring D-dimer concentrations for early detection of DVTs.

## Figures and Tables

**(a):** 

Case 1											
Day	1	2	3	4	5	6	7	8	9	10	11
PT (sec)	19.4	18.1	14.5	13.7	13.9	13.4	19.8	15.1	15.4	14.6	14.2
APTT (sec)	30.3	29.1	66.9	58.2	48.4	40.8	28.4	31.5	37.9	40.1	38.8
FDP (*μ*g/mL)	1.3	0.9	2.2	3	4.7	4.7	4.9	6.7	7.7	6.7	11.6
D-dimer (*μ*g/mL)	0.4	0.55	0.75	1.08	1.72	1.57	1.82	2.55	3.05	2.43	5.2
BUN (mg/dL)	10.4	3.1	4.1	5.1	8.8	13.4	26.8	25.1	29.8	25.4	25
SCr (mg/dL)	0.69	0.38	0.61	0.55	0.47	0.52	0.52	0.57	0.52	0.53	0.54
CK (U/L)	40	44	34	56	651	186	298	253	792	398	344
CRP (mg/dL)	0.01	0.03	7.85	12.77	18.9	11.28	2.54	3.38	9.98	10.95	11.26
Li (mEq/L)	3.95	0.99	0.53	0.27	<0.1	—	—	<0.1	—	—	—

**(b):** 

Case 2						
Day	-8	-1	1	2	5	7
PT (sec)	—	—	11.8	12.1	12	12
APTT (sec)	—	—	25.2	24.4	22.3	24.5
FDP (*μ*g/mL)	—	—	—	35.5	41.7	31.7
D-dimer (*μ*g/mL)	—	—	—	18.08	19.52	15.56
BUN (mg/dL)	—	—	15.7	11.7	17.2	—
SCr (mg/dL)	—	—	1.03	0.9	0.91	0.83
CK (U/L)	—	—	17	16	21	19
CRP (mg/dL)	—	—	—	0.21	0.03	—
Li (mEq/L)	1.92	1.5	0.98	0.59	—	—

Day 1: date of hospitalization; APTT: activated partial thromboplastin time; BUN: blood urea nitrogen; CK: creatine kinase; CRP: C-reactive protein; FDP: fibrinogen and fibrin degradation products; Li: lithium; PT: prothrombin time; SCr: serum creatinine.

## Data Availability

The data used to support the findings of this study are included within the article.
